# Obstructed Right Paraduodenal Hernia: An Unusual Cause of Small Bowel Gangrene

**DOI:** 10.7759/cureus.69192

**Published:** 2024-09-11

**Authors:** Dimpy Shah, Nitin Borle, Sachin Suryawanshi

**Affiliations:** 1 General Surgery, Topiwala National Medical College and Bai Yamunabai Laxman Nair Charitable Hospital, Mumbai, IND

**Keywords:** internal hernia, right paraduodenal hernia, small bowel obstruction, small bowel resection, superior mesentric artery

## Abstract

Paraduodenal hernias, especially right sided, are extremely rare entities that are difficult to diagnose due to their uncommon presentation. Some may experience small bowel obstruction. Such patients will have a guarded prognosis if complicated by sepsis due to strangulation or perforation. Hereby, we present a case report of a 15-year-old male who presented with a three-hour history of severe colicky abdominal pain. A computed tomography scan revealed acute small bowel obstruction. On laparotomy, it was found that the majority of the small bowel loops were obstructed in a sac on the right side of duodenojejunal flexure and had become gangrenous, suggesting strangulated right paraduodenal hernia. Due to extensive small bowel resection, this patient succumbed in the postoperative period. This case demonstrates how complex the diagnosis and management of this entity are, as well as how fatal the outcomes can be. Early diagnosis and surgical intervention remain the mainstay of treatment.

## Introduction

Paraduodenal hernias are a form of internal hernia believed to arise from a congenital defect that affects the retroperitoneal fixation of the mesentery, which is linked to the irregular rotation of the midgut [1]. These hernias can be categorized as left-sided or right-sided, with left-sided hernias being more prevalent at 75%, compared to 25% for right-sided hernias [1]. Their incidence still remains unclear as they are usually asymptomatic and mostly diagnosed during autopsy. Autopsy data indicate that the occurrence of internal hernias ranges from 0.2% to 0.9%, with paraduodenal hernias accounting for approximately 53% of these cases [2]. Although frequently asymptomatic, paraduodenal hernias can result in intermittent abdominal pain, nausea, and, in severe situations, bowel obstruction [3]. In this report, we present the case of a 15-year-old male who exhibited obstructive symptoms due to a right paraduodenal hernia.

## Case presentation

A 15-year-old male came to the casualty with a history of severe abdominal pain for three hours. He experienced cramping-like pain, which was accompanied by eight to nine episodes of non-bilious vomiting that contained food particles. Since the last six months, the patient was having intermittent low-intensity pain that would resolve on its own, so he had not taken any medical advice for the same. The patient denied any alterations in bowel or bladder habits and had no significant medical or surgical history. The patient was in persistent pain that was not settling even with analgesics. The abdomen was tender, guarded, and rigid. Bowel sounds were sluggish, and the digital rectal examination was unremarkable. Laboratory investigations revealed anemia and deranged creatinine (Table 1).

**Table 1:** Important blood parameters on admission.

Blood parameter	Result	Reference range
Hemoglobin	10.4 g/dL	13.5-17.5 g/dL
White blood cell count	7,500 cells/mm^3^	4,000-11,000 cells/mm³
Platelet count	212,000 cells/mm^3^	150,000-450,000 cells/mm³
Creatinine	2.0 mg/dL	0.6-1.3 mg/dL
Sodium	135 mEq/L	135-145 mEq/L
Potassium	4.8 mEg/L	3.5-5.0 mEq/L

**Figure 1:**
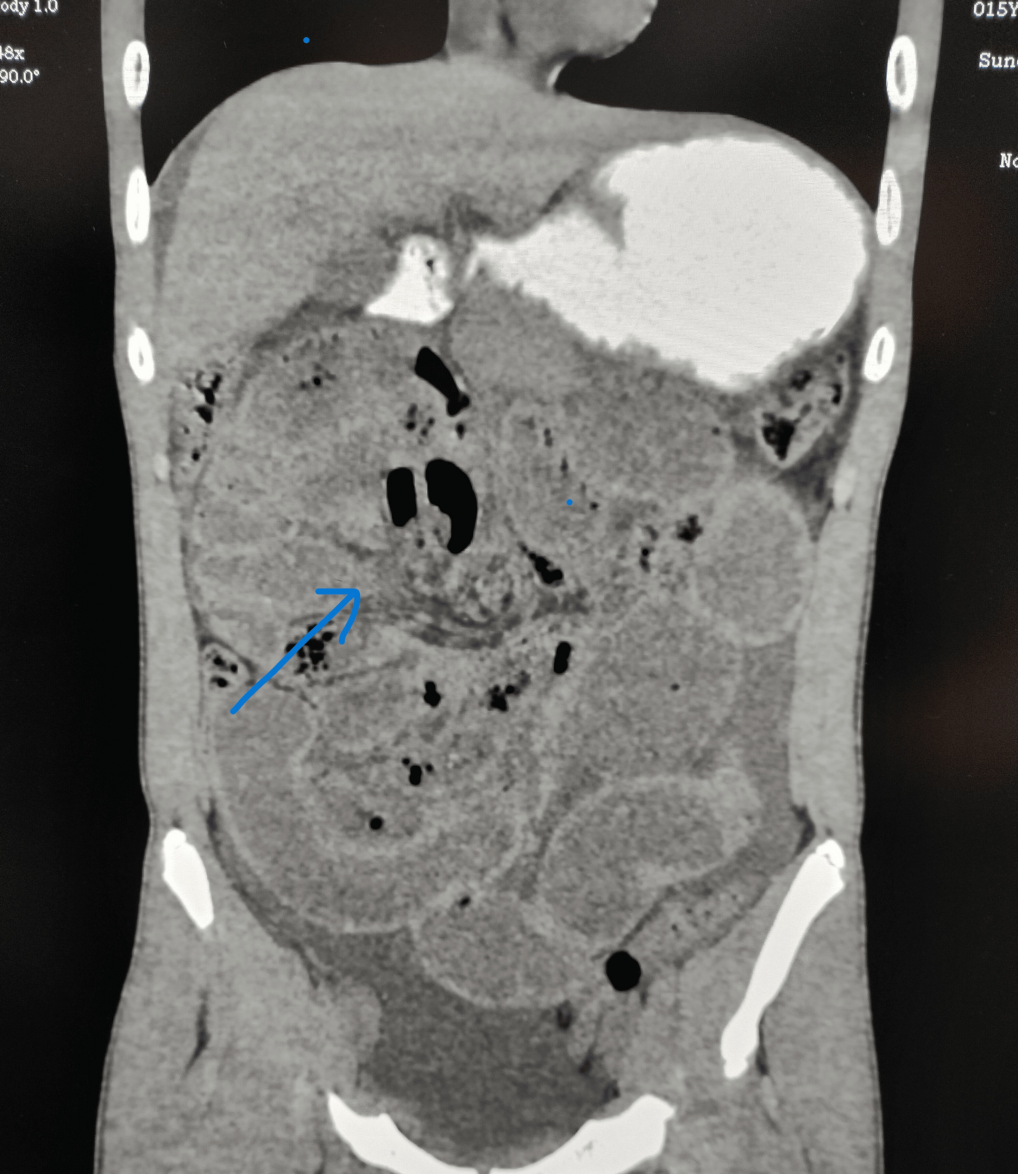
Plain computed tomography scan of abdomen and pelvis. The scan shows a sac-like structure in the right upper quadrant with entrapment of small bowel loops and twisting of vessels at the neck of the sac along with displacement of the duodenum (blue arrow).

**Figure 2:**
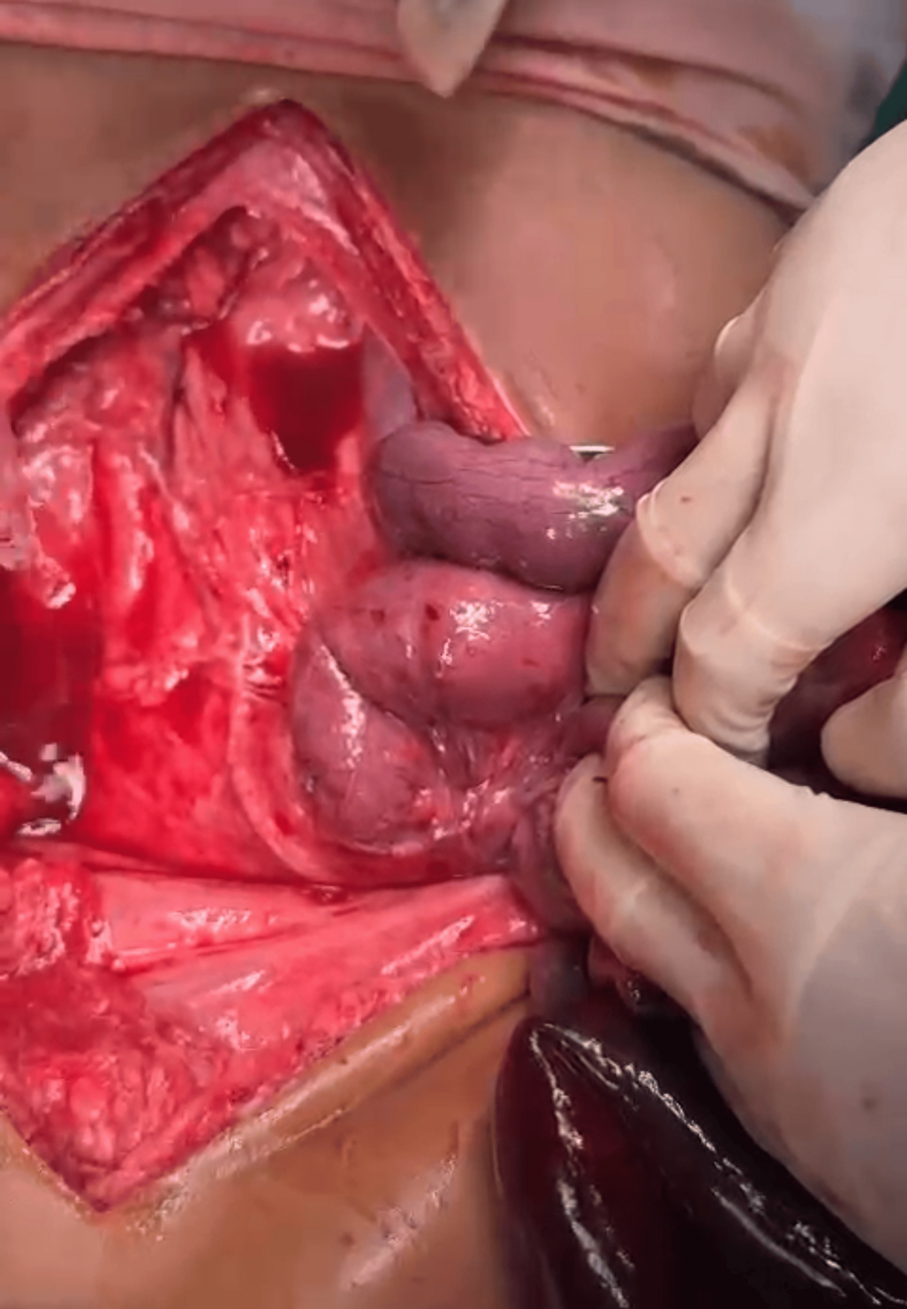
Intraoperative photo 1. The right side of the duodenojejunal flexure has a sac-like structure where the small bowel loops have herniated.

**Figure 3:**
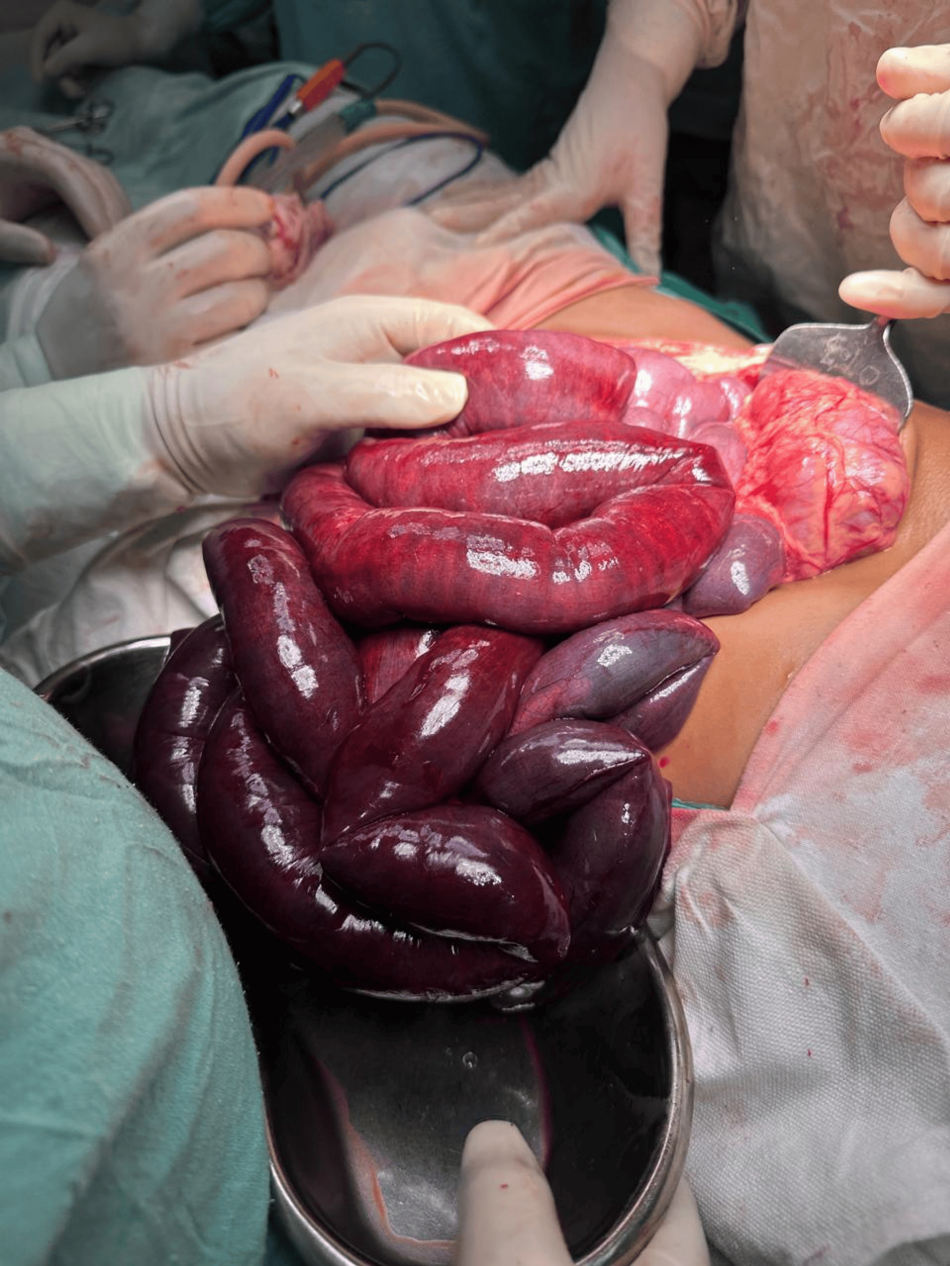
Intraoperative photo 2. Gangrenous small bowel loops were removed from the right paraduodenal hernia sac.

The plain abdominal radiograph was inconclusive. The plain computed tomography scan revealed gross dilatation of the small bowel loops with a maximum diameter measuring 4.42 cm with a transition point in the distal ileal loops 8 cm to 10 cm proximal to the ileocaecal junction along with moderate free fluid in the abdomen (Figure 1). Prior to surgery, written informed consent was taken from the patient's legal guardians. On laparotomy, it was found that the small bowel loops were obstructed inside a sac to the right of the superior mesenteric artery (Figure 2). The gangrenous changes started 50 cm distal to the duodenojejunal flexure up to 10 cm proximal to the ileocaecal junction (Figure 3).

The patient was thus diagnosed to have a right paraduodenal hernia. The gangrenous small bowel loops were retrieved by carefully relieving the constriction at the neck of the sac, not causing damage to any major vessels. After resecting the gangrenous small bowel loops, a double barrel stoma was made in the right iliac region. Despite all resuscitative efforts, the patient succumbed in the postoperative period due to sepsis.

## Discussion

This case clearly elucidates the complexities of diagnosing paraduodenal hernias as well as how fatal they can be. Our case is one of the few cases that needed small bowel resection due to small bowel strangulation [1].

Paraduodenal hernias are an uncommon cause of intestinal obstruction, representing approximately 0.5% of all hernias [1]. Right-sided paraduodenal hernias are significantly less common than their left-sided counterparts and occur through a defect in the jejunal mesentery known as Waldeyer's fossa, located at the third portion of the duodenum, posterior to the superior mesenteric artery [1]. They are more frequently observed in males, with a ratio of 2:1, and can occur in individuals of any age group [4]. These hernias typically present with nonspecific symptoms, such as nausea, vomiting, and intermittent abdominal cramping [1]. About 50% of patients with paraduodenal hernias may experience acute bowel obstruction, with a mortality rate that can reach up to 50% [4].

Contrast-enhanced computed tomography scan has a good specificity in diagnosis [1]. A right paraduodenal hernia is characterized by an unusual clustering of dilated small bowel loops within a sac-like structure located in the right upper abdomen [4]. The third portion of the duodenum is positioned above these dilated loops, while the root of the small bowel mesentery is situated in front [4]. The superior mesenteric vessels are found on the anteromedial edge of the fossa, and the right colic vein is shifted anteriorly [4]. In cases of intestinal malrotation, the normal appearance of the third portion of the duodenum crossing from right to left is absent, and the superior mesenteric vein is seen to the left of the superior mesenteric artery [4]. When obstruction occurs, additional findings such as dilated small bowel loops, air-fluid levels, mesenteric congestion, and fat stranding may be evident [4].

Surgery remains the primary treatment method, which may involve laparoscopy or exploratory laparotomy [1]. It is crucial to repair internal hernias promptly [1], as they can often go undetected until bowel compromise becomes severe, as seen in our case. Careful dissection during surgery is essential to avoid damaging major vessels, particularly the superior mesenteric artery, which supplies most of the small bowel and ascending colon [1]. In rare instances of small bowel gangrene, small bowel resection becomes necessary.

## Conclusions

Paraduodenal hernias, being rare entities, would never be looked upon as a probable diagnosis when a patient with abdominal pain presents to the emergency department. However, it should always be at the back of a surgeon’s mind in case of unexplainable abdominal pain with an inconclusive radiograph with a tender abdomen. Due to the rare nature of this entity, sometimes even radiologists may miss it on computed tomography scanning. Early diagnosis and intervention remain the mainstay of treatment.
